# Supplementary material for: Evaluation of Adjuvant Treatments for Adenoid Cystic Carcinoma of the Breast: A Population-Based, Propensity Score Matched Cohort Study from the SEER Database
Source: Diagnostics (Basel). 2022 Jul 21;12(7):1760. doi: 10.3390/diagnostics12071760 (PMC9324850; doi:10.3390/diagnostics12071760)
Supplement: Supplementary file 1 [file diagnostics-12-01760-s001.zip › Supplementary Table S1.pdf]

Table S1. Clinic and pathological characteristics of patients with ACC of the breast (N=399) stratified by adjuvant chemotherapy (without vs. with) before and after PSM.

Characteristic	Before PSM				After PSM			
	Without N=345	With N=54	P-value	SMD	Without N=41	With N=41	P-value	SMD
Age(%)								
<=60	172(49.9)	35(64.8)	0.058	0.306	24(58.5)	27(65.9)	0.649	0.151
>60	173(50.1)	19(35.2)			17(41.5)	14(34.1)		
Year of diagnosis(%)								
1975-2009	181(52.5)	35(64.8)	0.122	0.253	28(68.3)	25(61.0)	0.644	0.153
2010-2019	164(47.5)	19(35.2)			13(31.7)	16(39.0)		
Histology Grade(%)								
I	171(49.6)	15(27.8)	0.006	0.481	15(36.6)	15(36.6)	1.000	<0.001
II	122(35.4)	24(44.4)			17(41.5)	17(41.5)		
III-IV	52(15.1)	15(27.8)			9(22.0)	9(22.0)		
Tumor stage(%)								
T1	207(60.0)	24(44.4)	0.045	0.315	19(46.3)	21(51.2)	0.825	0.098
T2-T4	138(40.0)	30(55.6)			22(53.7)	20(48.8)		
Nodal status(%)								
Negative	340(98.6)	40(74.1)	<0.001	0.762	40(97.6)	40(97.6)	1.000	<0.001
Positive	5(1.4)	14(25.9)			1(2.4)	1(2.4)		
TNM stage(%)								
I	206(59.7)	21(38.9)	0.006	0.426	18(43.9)	21(51.2)	0.658	0.147
II-III	139(40.3)	33(61.1)			23(56.1)	20(48.8)		
Hormone receptor*(%)								
Negative	273(79.1)	43(79.6)	1.000	0.012	33(80.5)	33(80.5)	1.000	<0.001
Positive	72(20.9)	11(20.4)			8(19.5)	8(19.5)		
Surgery(%)								
BCS	234(67.8)	36(66.7)	0.990	0.025	28(68.3)	28(68.3)	1.000	<0.001
Total mastectomy	111(32.2)	18(33.3)			13(31.7)	13(31.7)		
Adjuvant radiotherapy(%)								
Without	182(52.8)	20(37.0)	0.045	0.320	18(43.9)	17(41.5)	1.000	0.049
With	163(47.2)	34(63.0)			23(56.1)	24(58.5)		

*ER positive and/or PR positive was categorized as HR positive. ER negative and PR negative was categorized as HR negative. Abbreviation:BCS=breast conserving surgery; PSM=propensity scores matching; SMD=standardized mean difference; TNM=tumor-node-metastasis.
