# Supplementary material for: Evaluation of Adjuvant Treatments for Adenoid Cystic Carcinoma of the Breast: A Population-Based, Propensity Score Matched Cohort Study from the SEER Database
Source: Diagnostics (Basel). 2022 Jul 21;12(7):1760. doi: 10.3390/diagnostics12071760 (PMC9324850; doi:10.3390/diagnostics12071760)
Supplement: Supplementary file 1 [file diagnostics-12-01760-s001.zip › Supplementary Table S2.pdf]

Table S2. Clinic and pathological characteristics of patients with ACC of the breast (N=399) stratified by adjuvant radiotherapy (without vs. with) before and after PSM.

Characteristic	Before PSM				After PSM			
	Without N=202	With N=197	P-value	SMD	Without N=79	With N=79	P-value	SMD
Age(%)								
<=60	97(48.0)	110(55.8)	0.144	0.157	44(55.7)	52(65.8)	0.254	0.209
>60	105(52.0)	87(44.2)			35(44.3)	27(34.2)		
Year of diagnosis(%)								
1975-2009	112(55.4)	104(52.8)	0.666	0.053	42(53.2)	39(49.4)	0.750	0.076
2010-2019	90(44.6)	93(47.2)			37(46.8)	40(50.6)		
Histology Grade(%)								
I	98(48.5)	88(44.7)	0.540	0.111	41(51.9)	47(59.5)	0.468	0.197
II	74(36.6)	72(36.5)			24(30.4)	23(29.1)		
III-IV	30(14.9)	37(18.8)			14(17.7)	9(11.4)		
Tumor stage(%)								
T1	113(55.9)	118(59.9)	0.484	0.080	47(59.5)	49(62.0)	0.871	0.026
T2-T4	89(44.1)	79(40.1)			32(40.5)	30(38.0)		
Nodal status(%)								
Negative	195(96.5)	185(93.9)	0.319	0.123	79(100.0)	77(97.5)	0.477	0.228
Positive	7(3.5)	12(6.1)			0(0.0)	2(2.5)		
TNM stage(%)								
I	110(54.5)	117(59.4)	0.371	0.100	47(59.5)	48(60.8)	1.000	0.026
II-III	92(45.5)	80(40.6)			32(40.5)	31(39.2)		
Hormone receptor* (%)								
Negative	153(75.7)	163(82.7)	0.110	0.173	57(72.2)	54(68.4)	0.728	0.083
Positive	49(24.3)	34(17.3)			22(27.8)	25(31.6)		
Surgery(%)								
BCS	86(42.6)	184(93.4)	<0.001	1.299	69(87.3)	68(86.1)	1.000	0.037
Total mastectomy	116(57.4)	13(6.6)			10(12.7)	11(13.9)		
Adjuvant chemotherapy(%)								
Without	182(90.1)	163(82.7)	0.045	0.216	75(94.9)	71(89.9)	0.368	0.192
With	20(9.9)	34(17.3)			4(5.1)	8(10.1)		

*ER positive and/or PR positive was categorized as HR positive. ER negative and PR negative was categorized as HR negative.

Abbreviation:BCS=breast conserving surgery; PSM=propensity scores matching; SMD=standardized mean difference; TNM=tumor-node-metastasis.
